# Dynamic machine learning approach for workload prediction in cloud environments

**DOI:** 10.1038/s41598-026-40777-z

**Published:** 2026-04-01

**Authors:** Mona Nashaat, Walid Moussa, Rawya Rizk, Walaa Saber

**Affiliations:** Electrical Engineering Department, Faculty of Engineering, Port Said University, Port Said, 42526 Egypt

**Keywords:** Machine learning, Cloud computing, Container, Time series, Workload prediction, Computer science, Information technology

## Abstract

Containers, as a lightweight technology for virtualizing applications, have recently revolutionized the management of cloud applications. Containers can be scaled up or down rapidly and easily depending on the workload. Predicting workload is crucial for efficiently auto-scaling of resources in cloud environments. Accurate predictions help estimate the number of needed containers, which reduces costs and ensures optimal resource utilization. However, workloads for web applications often vary across different applications and timeframes. A single prediction model struggles to capture these diverse workload patterns, highlighting the need for more specialized or adaptive models to efficiently handle the dynamic nature of cloud application workloads. To address this limitation, this paper introduces a **M**onitor–**T**rain–**T**est–**D**eploy (MTTD) framework, a closed-loop workload-prediction system that continuously retrains, evaluates, and selects the most suitable predictive model during runtime based on observed workload behavior and recent prediction errors. Instead of relying on one static predictor, MTTD dynamically switches among multiple learning models to maintain stable forecasting quality under changing workload conditions. Experiments conducted on containerized web-application workloads show that the proposed framework improves prediction accuracy by up to 15% compared with individual fixed models, while also reducing performance degradation during model transitions, leading to more reliable container provisioning and lower resource waste.

## Introduction

Cloud computing has become a core platform for hosting and delivering modern digital services, enabling organizations to store, process, and serve large volumes of data with elastic resource allocation^1–3^. By allowing computing resources to be scaled on demand, cloud platforms can respond to fluctuating workloads without requiring permanent over-provisioning. Efficient use of these resources depends heavily on load balancing mechanisms that distribute incoming requests across available servers to avoid congestion and reduce response time^4–6^. As a result, maintaining balanced workloads across cloud nodes is essential for ensuring service quality and controlling operational costs in large-scale cloud systems.

Container-based virtualization has further transformed cloud application deployment by providing lightweight, portable execution environments that package applications together with their dependencies^7^. Unlike traditional virtual machines, containers share the host operating system, reducing overhead and enabling faster startup and higher deployment density^8,9^. This makes containers particularly suitable for applications that require frequent scaling and rapid response to workload changes. By isolating applications at the process level, container platforms also improve reliability, as failures in one container do not propagate to others^10^. These characteristics have made containers a key enabler of modern microservice-based cloud architectures.

To evaluate and compare resource management and autoscaling strategies under controlled conditions, cloud simulation tools are widely used to emulate large-scale cloud environments without the cost and complexity of physical deployments^11^. Among these tools, CloudSim provides a flexible framework for modeling data centers, virtual machines, workloads, and provisioning policies^12^. Several extensions have been developed to enhance its support for containerized systems, energy-aware computing, and network modeling, making it suitable for studying dynamic cloud environments and testing new scheduling and scaling approaches.

Accurate prediction of server workloads is a key requirement for efficient operation of web-based cloud services^13^. By estimating future resource demand, forecasting models enable proactive scaling decisions that avoid both unnecessary over-provisioning and performance degradation caused by insufficient capacity. These predictions are typically derived from historical workload traces using statistical or learning-based methods. A wide range of such models has been explored in the literature, each offering different trade-offs in terms of accuracy, stability, and computational cost^14–17^.

A major difficulty in applying these models arises from the highly variable and non-stationary nature of cloud workloads^18,19^. Traffic patterns can change abruptly due to user behavior, time-dependent usage, promotional events, or software updates, leading to sudden and irregular load variations. Under these conditions, models trained on past data may become unreliable, causing either excessive resource allocation or delayed scaling responses. As a result, static prediction approaches often struggle to maintain consistent performance when workload characteristics evolve.

Predictive models are widely used to estimate future workload behavior, yet achieving consistently high accuracy remains challenging due to the variability of web traffic patterns^20,21^. Classical time-series methods such as the Autoregressive Integrated Moving Average (ARIMA) model have been applied to forecast server workloads because of their effectiveness in capturing trend and seasonality in historical data^22,23^. However, ARIMA relies on linear assumptions and therefore performs poorly when workload patterns exhibit strong non-linear behavior, which is common in HTTP traffic. Regression-based approaches, including linear and support vector regression, have also been explored, offering relatively simple and interpretable models while providing some capacity to model nonlinear relationships.

Neural-network-based approaches have gained increasing attention for workload forecasting because of their ability to learn complex temporal patterns from data. In particular, recurrent neural networks (RNNs) and their variants, such as Long Short-Term Memory (LSTM) networks, have shown strong performance in modeling long-range dependencies in time-series workloads^24–26^. These models are well suited to capturing irregular traffic behavior commonly observed in cloud applications. Hybrid approaches that combine statistical models, such as ARIMA, with neural networks have also been proposed to exploit both linear trend modeling and nonlinear pattern learning. While such hybrid and deep-learning-based methods often achieve higher accuracy, they typically introduce substantial computational overhead and increased training complexity.

Moreover, hybrid models that integrate multiple predictors simultaneously are often resource-intensive and difficult to maintain in operational cloud environments^27^. At the same time, single-model approaches are usually tuned to specific workload traces, limiting their ability to generalize across different applications and deployment settings. This trade-off between model complexity and adaptability highlights the need for a lightweight and adaptive prediction framework that can respond effectively to diverse and evolving workload patterns without high computational cost.

The proposed **M**onitor–**T**rain–**T**est–**D**eploy (MTTD) framework is designed for deployment in operational cloud environments and can be integrated with container orchestration platforms such as Kubernetes or Docker Swarm together with existing monitoring infrastructures in public and private data centers. By continuously evaluating prediction performance under changing workload conditions, MTTD enables adaptive, prediction-driven resource management for containerized applications.

The MTTD framework provides the following capabilities:

- It supports dynamic selection among multiple prediction models, allowing the system to switch between different algorithmic approaches based on their observed performance under current workload conditions.
- It enables the use of models from different methodological families (e.g., statistical and learning-based) without combining them simultaneously, thereby avoiding the computational overhead of hybrid predictors.
- It maintains forecasting stability by ensuring that the most accurate available model is used at each stage of operation.
- It allows new prediction models to be added and poorly performing ones to be removed during runtime as workload behavior evolves.

The remainder of this paper is organized as follows. Section  2 reviews related work on container-based cloud systems and workload prediction. Section  3 presents the design and operation of the proposed Monitor–Train–Test–Deploy framework. Section  4 describes the experimental setup, datasets, evaluation metrics, and performance results. Section  5 concludes the paper and outlines directions for future work.

## Related work

A wide range of workload prediction methods has been proposed for cloud and containerized environments, spanning statistical models, neural networks, and hybrid approaches. Ensemble-based techniques such as FAST^28^ combine sliding-window analysis with multiple local predictors to model both long-term trends and short-term fluctuations in large-scale cluster traces.

Similarly, deep-learning-based forecasting has been widely explored due to its ability to model non-linear and temporal dependencies in workload traces. Approaches such as CrystalLP^29^ and Bi-LSTM-based hybrid models^30^ apply recurrent networks to capture long-range dependencies in storage and CPU workload time series, demonstrating improved prediction accuracy compared with classical methods such as ARIMA and standard LSTM. However, these models rely heavily on extensive preprocessing, bidirectional learning, or multiple network components, which introduces substantial computational and memory overhead and limits their suitability for large-scale or time-sensitive environments.

Hybrid prediction models have also been proposed to exploit the complementary strengths of statistical and learning-based techniques. ARIMA–LSTM and ARIMA–ANN models^31,32^ combine linear trend modeling with non-linear pattern learning, while collaborative filtering has been incorporated to exploit correlations between servers^31^. Although these hybrid systems often outperform standalone predictors on specific datasets, they require simultaneous execution of multiple models and careful parameter tuning, resulting in increased training time and reduced operational efficiency when workload behavior changes.

More complex deep architectures, including CNN–LSTM and DCNN–LSTM models^33,34^, have further improved prediction accuracy by extracting multi-scale features and temporal dependencies from workload traces. These models achieve low error rates and reduced SLA violations across Google, Alibaba, and BitBrain datasets. However, their reliance on deep convolutional and recurrent layers leads to high computational cost, making them difficult to deploy in real-time cloud control loops.

Other hybrid learning strategies, such as LSTM–SVR combinations^35^, aim to balance short-term responsiveness with long-term memory. While these approaches improve forecasting performance over individual models, they further increase system complexity and require careful coordination between multiple predictors.

Overall, existing workload-prediction methods face two persistent limitations. First, hybrid and deep models incur high computational and operational overhead, which reduces their practicality for real-time container scaling. Second, most models are optimized for specific datasets or workload profiles, limiting their ability to generalize across diverse cloud applications and deployment environments. A comparative summary of the reviewed methods is provided in Table 1. These limitations motivate the need for an adaptive framework that can select among multiple prediction models dynamically, rather than combining them simultaneously, in order to maintain accuracy while controlling computational cost.

**Table 1:** Summary of representative workload prediction approaches and their main characteristics reported in the literature.

Ref.	Approach/model	Prediction target	Key techniques	Environment/dataset	Key strengths	Limitations
^28^	FAST	Cloud workload	Ensemble model, adaptive sliding window, time locality	Google cluster traces	High accuracy for dynamic workloads	Focused on VM-level workloads, not container runtime
^29^	CrystalLP-based on Deep learning	Storage system workload	LSTM deep learning enhancement	Web Search Engine I/O	Using deep learning for accurate modeling of nonlinear temporal patterns in storage systems	Limited to storage systems
^30^	BHyPreC	VM CPU workload	Hybrid Bi-LSTM RNN LSTM	Bitbrain distributed data center	Accurate CPU usage prediction	VM-centric, lacks container-awareness
^31^	ARIMA-LSTM-CF	Backend server traffic	Statistical + hybrid traditional and deep learning	Bank real traffic datasets	Combines linear and nonlinear modeling	tailored for bank traffic data
^32^	Statistical hybrid ARIMA ANN model	Cloud CPU and Memory utilization	Time-series statistical models	Google’s 29-day trail and BitBrain (BB)	Lightweight and interpretable	Lower adaptability to dynamic workloads
^33^	DCNN–LSTM	Cloud workload	Deep CNN + LSTM	Google trace datasets	High accuracy on cloud computing nonlinear forecasting	High computational complexity
^34^	Hybrid pCNN–LSTM	CPU utilization	1D CNN + LSTM	Google cluster trace, Alibaba trace and Bitbrains data	Robust to workload variations	Does not consider autoscaling or containerization
^35^	LSTM–SVR	Traffic speed	Hybrid deep learning + SVR	PEMS-BAY traffic speed datasets	Effective short-term prediction	Application-specific, not cloud-focused

## Proposed solution

This section presents the proposed solution for workload prediction in cloud-based web applications. The framework is designed to support containerized services deployed in clustered cloud environments. First, the workload prediction problem is formulated, and typical workload patterns are described. The architecture of the proposed framework is then introduced.

### Problem formulation

Workload prediction in cloud environments can be modeled as a time-series forecasting problem, where future HTTP request rates are estimated based on historical observations over a fixed time window. The proposed framework continuously records workload measurements in a time-series database, where each data point is associated with a timestamp and stored in chronological order. This allows the system to maintain a complete historical record of workload behavior for model training and evaluation.

Time-series datasets can be classified as univariate or multivariate. A univariate time series contains observations of a single variable over time, while a multivariate series includes multiple variables measured at the same timestamps. In this study, a univariate workload series representing the number of HTTP requests is used to train the prediction models. After training, the resulting models are deployed to estimate the workload for the upcoming time window based on recent historical data.

**Fig. 1:**
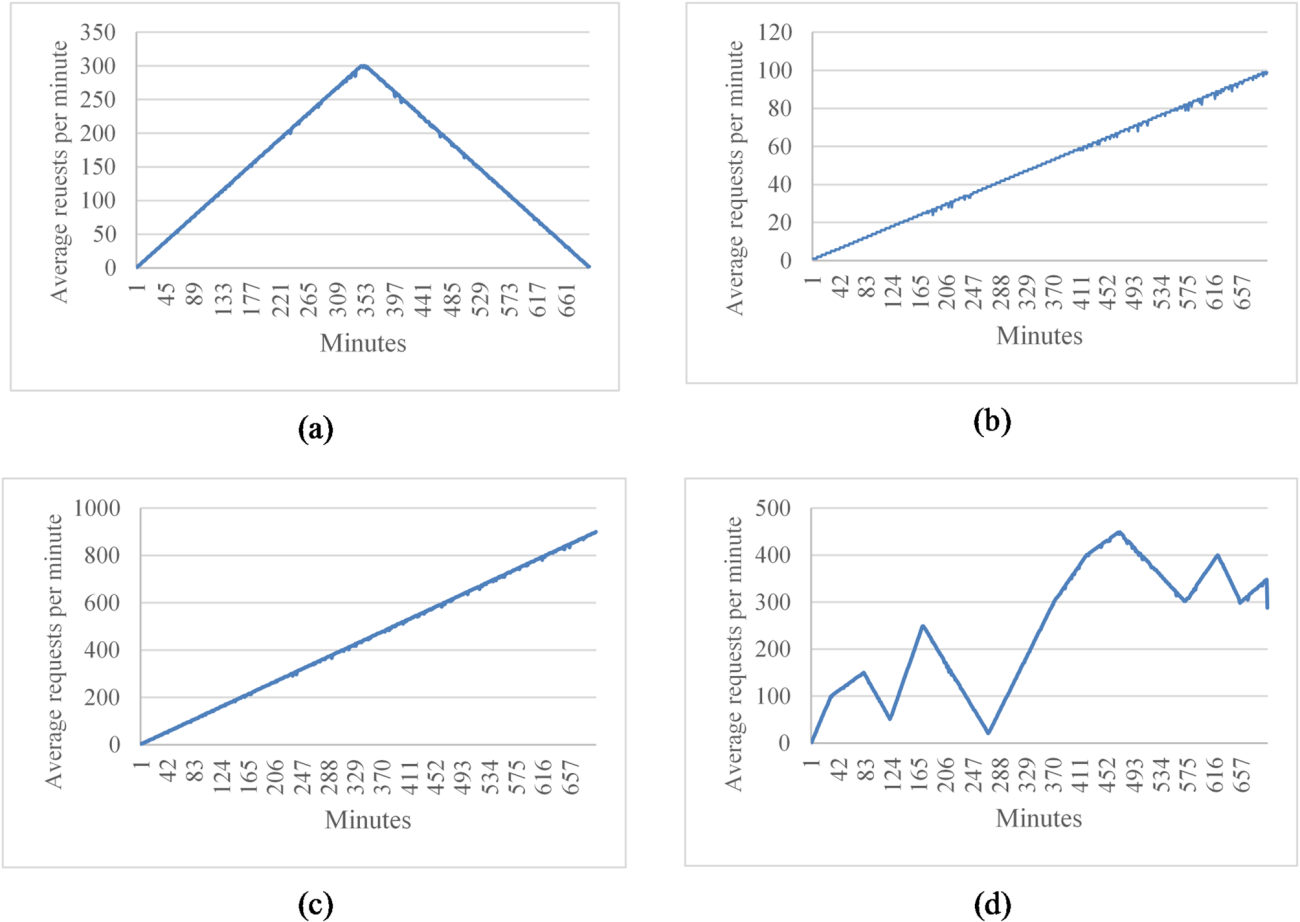
Workload patterns used in the experiments, including uniform, linear, high-rate linear, and random request distributions. (**a**) Workloads with normal distribution, (**b**) Workloads with change rate = 0.35 request/s per minute, (**c**) Workloads with change rate = 2.31 request/s per minute, (**d**) Workload random change rate.

The proposed framework is designed to operate under diverse workload conditions, where request rates vary over time according to user activity patterns. In particular, three representative workload patterns are considered, as illustrated in Fig. 1. Figure 1a shows a normal workload profile in which the request rate increases gradually, reaches a peak, and then decreases smoothly over time, following a roughly bell-shaped distribution.

Alternatively, Fig. 1b, c illustrate HTTP request workloads that exhibit continuous growth over time, but with different rates of change. In these patterns, the workload increases smoothly, and the request density does not vary abruptly. The two figures depict different growth rates of HTTP requests, resulting in distinct workload dynamics. The average workload change rate is defined as:

1 $$\:Avg\:rate=\:\frac{1}{N-1\:}\:{\sum\:}_{i=1}^{N-1}\:\:\left|{w}_{i+1}-{w}_{i}\right|$$

where *N* represents the number of time points, w_i_ and w_i+1_ represent workload at time *i*, *i + 1* respectively.

Figure 1d represents a random workload pattern in which the number of incoming requests changes unpredictably over time. In this case, the workload density varies sharply, making accurate prediction critical for enabling the system to react promptly and maintain stable performance.

### System architecture

**Fig. 2:**
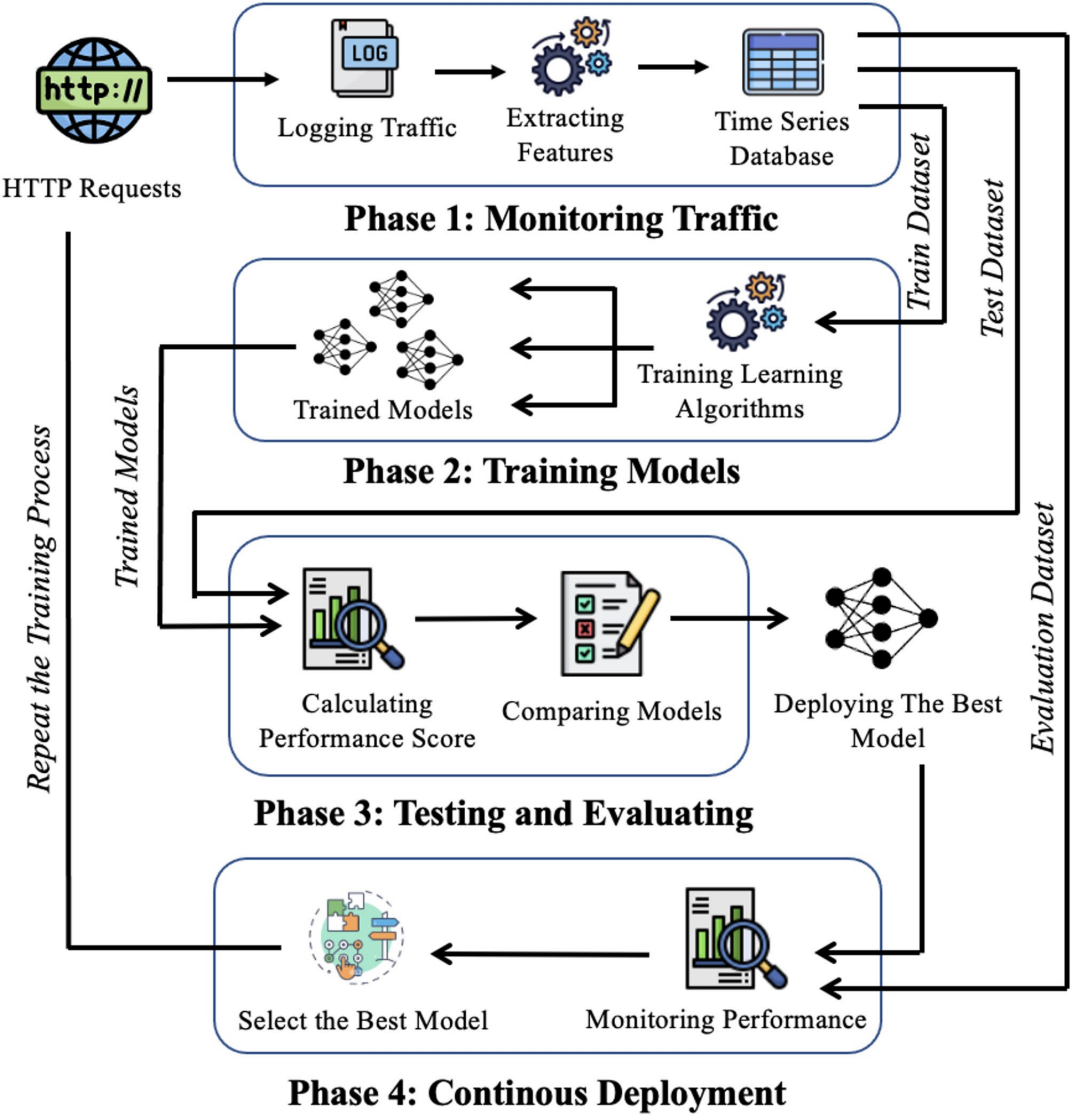
Overall architecture of the proposed framework.

The proposed framework is designed to predict future HTTP request volumes in cluster-based cloud environments. An overview of the system architecture is shown in Fig. 2. The framework operates as a closed-loop process consisting of four main phases: Monitoring, Training, Testing, and Deployment.

During the Monitoring phase, the system continuously observes the cloud environment and collects workload data from running containerized applications. This phase runs in parallel with the remaining stages to ensure that up-to-date workload information is always available.

In the Training phase, the collected time-series data are used to train a set of workload prediction models based on different learning algorithms. Each model learns to forecast the number of HTTP requests for the next time window.

The Testing phase evaluates the trained models using recent workload data to determine their prediction accuracy. Based on this evaluation, the framework identifies the model that best matches the current workload characteristics.

In the Deployment phase, the selected model is activated and used to generate workload predictions for the upcoming time window. The performance of the deployed model is then monitored continuously using newly observed data. If prediction accuracy degrades, the framework triggers retraining and model reselection; otherwise, the current model remains in use for the next window. The detailed operation of each phase is described in the following subsections.

#### Phase 1: monitoring traffic and collecting data

In this phase, incoming HTTP requests are collected and stored in a time-series database. HTTP requests form the basis of web communication and are used for various tasks, including loading web pages, submitting forms, fetching data from web APIs, and interacting with web services. Therefore, HTTP requests consist of several components, each serving a specific purpose in communication between the user and the web server. The main components of an HTTP request include the HTTP Method (e.g., GET, POST, etc.), the Uniform Resource Locator (URL), headers, the request body, and request parameters. The records are logged and stored on the server, with each request recorded with its corresponding timestamp. To prepare the data for training machine learning models, the collected raw data are converted into a format that the models can use. Hence, the framework aggregates the logged requests to create time-series datasets where each record represents the time in minutes and the average number of HTTP requests per second during that minute.

The traffic monitoring and data collection phase is an ongoing, dynamic process that plays a pivotal role in traffic prediction. This phase involves systematic and continuous observation, measurement, and recording of incoming requests. The significance of this continuous process cannot be overstated. It is the foundation for subsequent phases. By consistently gathering and reviewing data, we ensure that our framework remains informed and adaptive, enabling the algorithm to make informed decisions and maintain a proactive stance in traffic prediction.

#### Phase 2: training machine learning models for traffic prediction

During this phase, the framework retrieves recent workloads from the time-series dataset. This data is then split into two datasets: training and testing. Training and testing models on different datasets helps assess the quality of their pattern generalization to data they have not seen before. Therefore, the framework divides the collected data over a specific duration into training and testing datasets. During the training phase, the framework utilizes one main hyperparameter: the historical time window. This parameter represents the number of historical data records used to train the prediction models and denotes the size of the training dataset. Based on insights obtained from the experimental evaluation in Sect.  4, the time window is set to a default value of approximately 6 h. Although this value can be adjusted by the user, we found that data collected for 6 h is sufficient to build effective learning models for traffic prediction. The default value provides a representative sample of network behavior since it covers typical usage patterns and exhibits temporal variability. Moreover, longer data collection periods may require more storage capacity, computing power, and time, which may not be feasible or cost-effective.

The framework uses a training dataset to train various machine learning algorithms. It considers three algorithms: ARIMA, Support Vector Regression (SVR), and Random Forest Regressor (RF). ARIMA stands for *A*uto-*R*egressive *I*ntegrated *M*oving *A*verage, which is one of the most popular and in-demand time series forecasting algorithms. This algorithm is based on the idea that previous records in a time series dataset can be used to predict upcoming values. Similarly, SVR is another machine learning algorithm for regression analysis. It aims to approximate a function that describes the relationship between independent variables and the continuous dependent variable with minimum prediction error. To achieve this goal, SVR looks for a hyperplane in continuous space that best fits the data points. The input variables are mapped to a high-dimensional feature space, and the hyperplane that maximizes the margin between it and the nearest data points while minimizing prediction error is selected. Likewise, the Random Forest Regressor is an ensemble technique that combines numerous decision trees to determine the final output instead of relying on just one decision tree.

Although the framework is designed to easily accommodate other learning algorithms, the choice of these three algorithms—ARIMA, SVR, and Random Forest Regressor—is based on the characteristics of the collected data. Since the dataset for our problem is time series, ARIMA is well-suited for time series data, particularly when there is evidence of temporal dependencies and seasonality, which exist in network traffic data. The data in our case exhibit a common type of temporal dependency, which occurs when the current traffic volume is correlated with past traffic volumes at specific time lags. For example, if web users tend to access a particular website simultaneously every day, there will be a strong temporal dependency with a lag of 24 h. Likewise, seasonality refers to regular and predictable patterns that occur at fixed intervals over time. In network traffic data, seasonality typically represents recurring trends, such as daily, weekly, or monthly patterns. For instance, some websites or services may experience monthly or annual seasonality; online shopping websites may see surges in traffic during holiday seasons like Black Friday. Moreover, SVR and Random Forest are effective when dealing with non-linear relationships. Non-linear relationships can arise when multiple factors interact in complex ways to influence network traffic. For instance, the number of HTTP requests may not increase or decrease proportionally with a single input variable, such as time. Instead, interactions between time, user behavior, content popularity, and other variables can lead to non-linear patterns.

#### Phase 3: testing and model selection

After training the prediction models, the output models are tested using the test dataset obtained from the previous step. Based on the testing results, the framework selects the best model and uses it as the active model for the next deployment cycle. The framework considers two metrics to evaluate the accuracy of the trained models. First, it utilizes Root Mean Square Error (RMSE), which is the standard deviation of the residuals (prediction errors) and can be defined as follows:

2 $$\:RMSE\left({y}_{a},{y}_{p}\right)=\:\sqrt{\frac{1}{n}{\sum\:}_{i=1}^{n}\:\:{({y}_{a}-{y}_{p})}^{2}}$$

Similarly, the framework calculates the Mean Absolute Error (MAE), which is a measure of errors between predicted and actual values and is formally defined as^36,37^:

3 $$\:MAE\left({y}_{a},{y}_{p}\right)=\:\frac{1}{n}{\sum\:}_{i=1}^{n}\:\:{|y}_{a}-{y}_{p}|$$

where $\:{y}_{a}$, $\:{y}_{p}$, and *n* represent the actual workload, predicted workload, and number of samples used to evaluate the model, respectively. These metrics describe the deviation of predicted values away from actual values; lower metric values mean a better model. Then, the framework considers RMSE and MAE to calculate the performance score as described in the following equation:

4 $$\:{Score\:}_{Performance}=\:({w}_{1}*\frac{RMSE}{\sqrt{\frac{1}{n}{\sum\:}_{i=1}^{n}\:\:{{y}_{a}}^{2}}}+{w}_{2}*\frac{MAE}{\:\frac{1}{n}\:{\sum\:}_{i=1}^{n}\:\:{y}_{a}})*100$$

where $\:{w}_{1}$ and $\:{w}_{2}$ represent the weights for RMSE and MAE, respectively, as W_1_ + W_2_ = 1. The values of $\:{w}_{1}$ and $\:{w}_{2}$ can be alerted by the framework user according to the error accuracy with system workload behavior. However, both RMSE and MAE were found to have equal effects during the experiments. Therefore, their values are set to 0.5.

Score measure the average error prediction percentage relative to actual values, in case of ideal case of no error prediction score will be zero. When average prediction error more than the average actual value Score will exceed 100%.

In (4), RMSE is divided by the root mean square of actual values, and MAE is divided by the mean of actual values as a sort of normalization. This is because the range of actual values affects the range of MAE and RMSE values, which may give a deceptive indication of whether the algorithm’s prediction performance is increasing or decreasing. Table 2 presents the parameter notations used in the proposed model.

**Table 2:** Notation and definitions of the parameters used in the proposed framework.

Parameter	Meaning
*RMSE*	Root mean square error
*MAE*	Mean absolute error
_*n*_	Number of samples
*y*_*a*_	Actual workload
*Y*_*p*_	Predicted workload
*Score*	The average error prediction
*w*_*1*_	Score weight for RMSE
*w*_*2*_	Score weight MAE
*Threshold*	Measure performance of algorithm, score shouldn’t exceed third value

#### Phase 4: continuous deployment

After selecting the best model, it is utilized as the active model for the next deployment cycle. Hence, it is deployed for traffic prediction. The framework then applies a phase of continuous monitoring in which the model in the production environment is periodically evaluated to ensure it is providing accurate predictions. When the framework notices model drift or performance degradation, it implements alerts and triggers the retraining phase.

To implement the concept of continuous deployment, the framework automates the deployment of machine learning models into production environments in a seamless and continuous manner. The framework considers a deployment cycle in which the model predictions are used to estimate network traffic. At the same time, the framework uses the real traffic monitored during the first phase. Then, the model is evaluated by the end of each deployment cycle, and the metrics are collected to detect model issues. The primary goal of continuous deployment in the context of machine learning is to enable rapid and reliable model updates and improvements while minimizing human intervention and reducing deployment risks. Therefore, the framework utilizes another hyperparameter called the retraining threshold to control the degradation in the model performance after deployment. So, the framework triggers the retraining phase when the difference between the predicted values and the real workloads exceeds the threshold in two successive deployment cycles.

Moreover, the value of the retraining threshold gradually changes according to the degree of performance degradation. For example, the score is first set as:

5 $$\:{Threshold}_{t+1}\:\:=\:{Score\:}_{t}+\left(\:{l}_{1\:}*\:{Score\:}_{t}\right)$$

When the model score exceeds the threshold for the first time, the value is then adapted as:

6 $$\:{Threshold}_{t+2}=\:{Score\:}_{t+1}+{(\:l}_{2}*\:{Score\:}_{t+1})$$

where $\:{Threshold}_{t+1}\:and\:{Threshold}_{t+2}$ are threshold for two successive deployment cycles. $\:\:\:{Score\:}_{t}$ and $\:{Score\:}_{t+1}$ are the performance score of active algorithms during the test cycle and first deployment cycle respectively. $\:{l}_{1}$ and $\:{l}_{2}$ are the weights during the continuous deployment phase that control the framework tolerance toward performance degradation. The default values of $\:{l}_{1}$ and $\:{l}_{2}$ are set to 0.75 and 0.5, respectively, to implement the error amplification concept. For traffic prediction, the system should be less forgiving of errors or deviations from the expected behavior as those errors become more pronounced or more frequent. Error amplification can lead to cascading failures and increased system instability, making it a critical consideration in the design and reliability of the framework.

Therefore, when the model score degrades, exceeding the threshold during two successive deployment cycles, the framework utilizes these insights to make informed decisions about retraining models with newly collected data. Since the data in real-world applications is dynamic, patterns, trends, and relationships can change over time. By retraining models with new data, they can adapt to these evolving patterns and continue to provide accurate predictions or classifications.

**Fig. 3:**
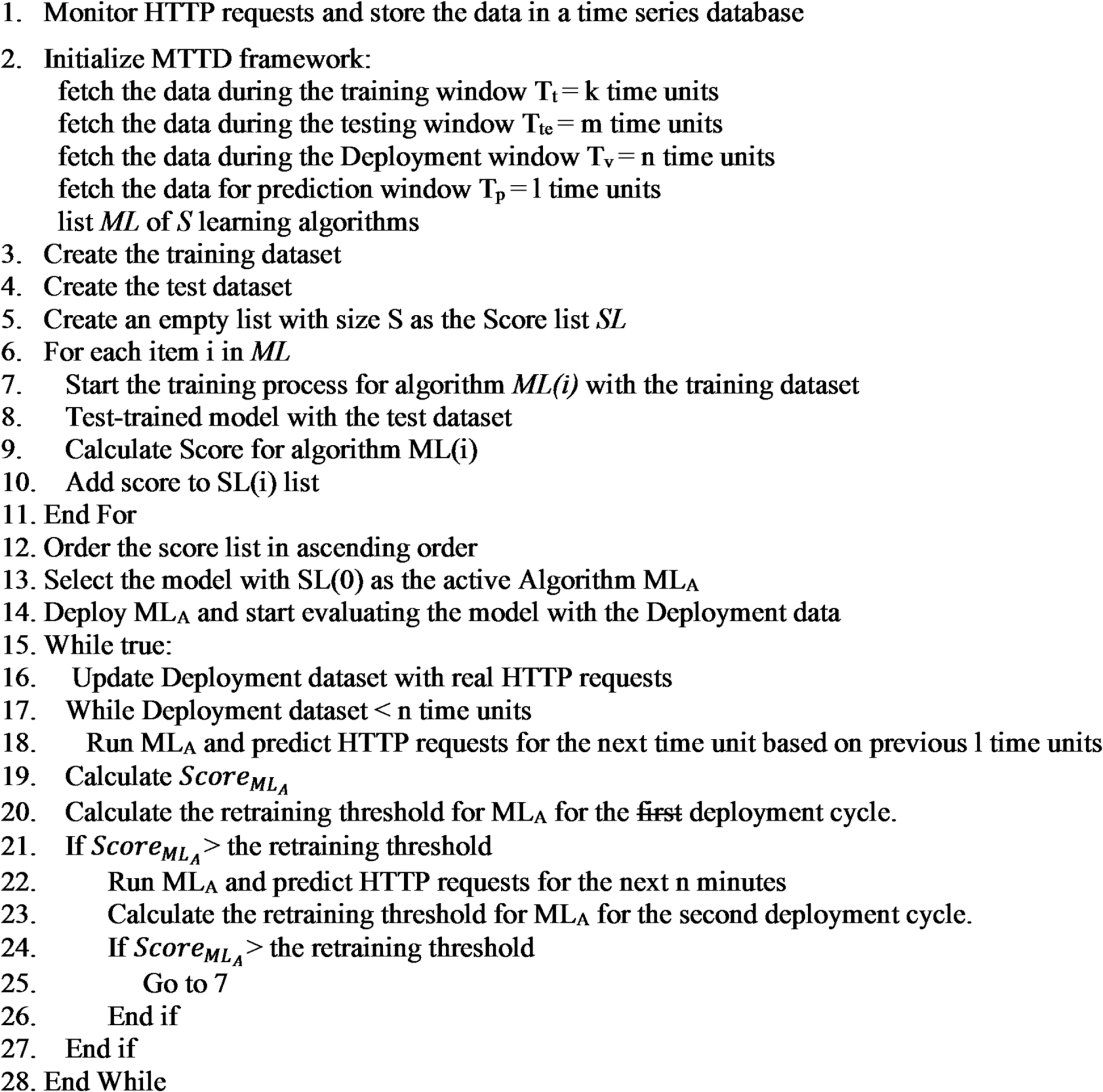
Algorithmic workflow of the proposed MTTD framework.

**Fig. 4:**
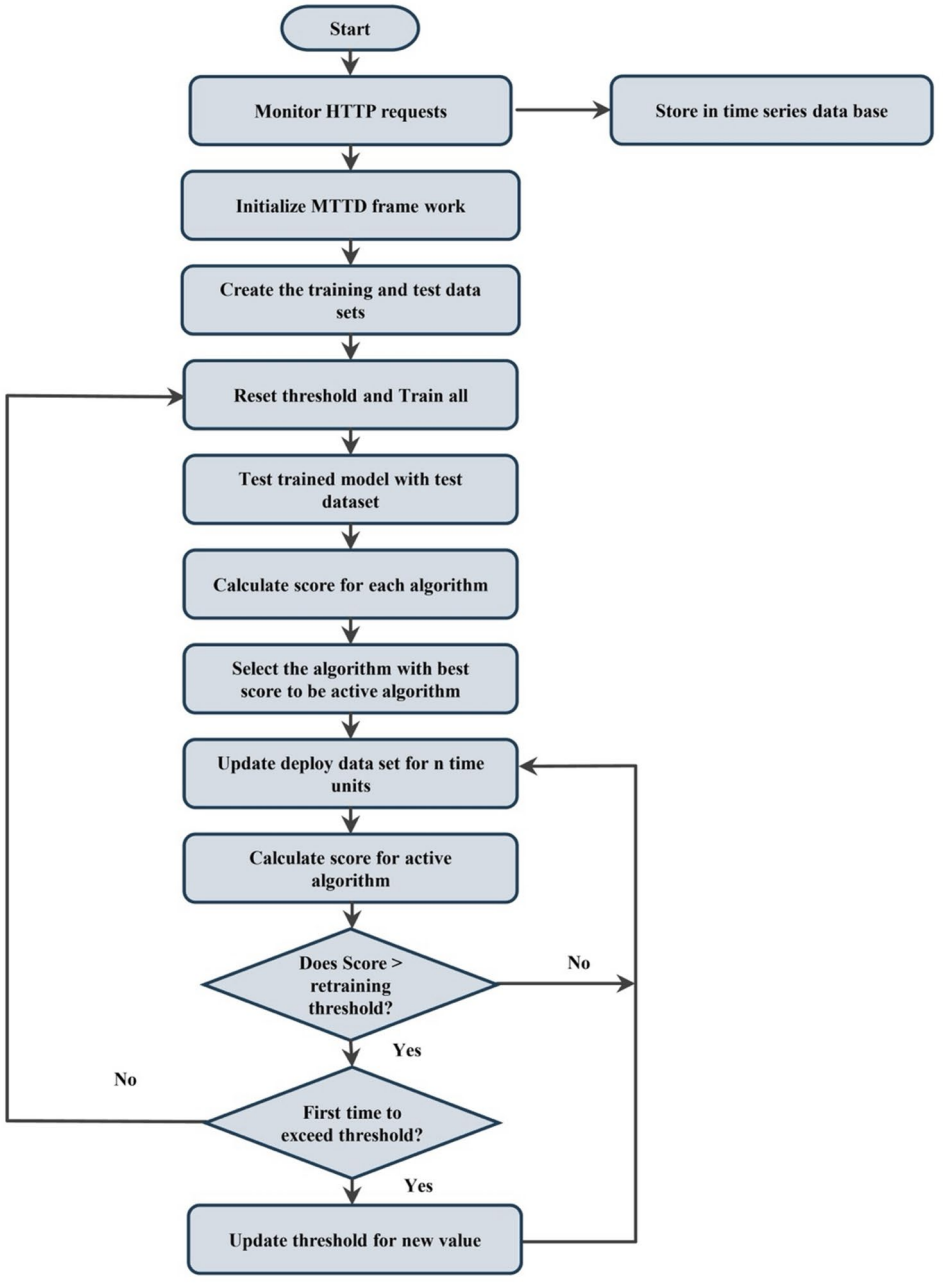
Flowchart of the operational process of the proposed framework.

The computational overhead of the proposed framework is kept low because only one prediction model is active at any time, while the monitoring and model-selection processes operate with minimal resource usage. Model switching and retraining are initiated only when prediction performance drops below predefined thresholds, ensuring that computational resources are used efficiently in operational cloud environments. The overall workflow of the proposed framework is illustrated in Fig. 3, while a flowchart describing the complete system operation is shown in Fig. 4.

## Experimental evaluation

This section describes the experimental setup used to evaluate the proposed framework and presents the results of the empirical analysis.

### Simulation environment

**Fig. 5:**
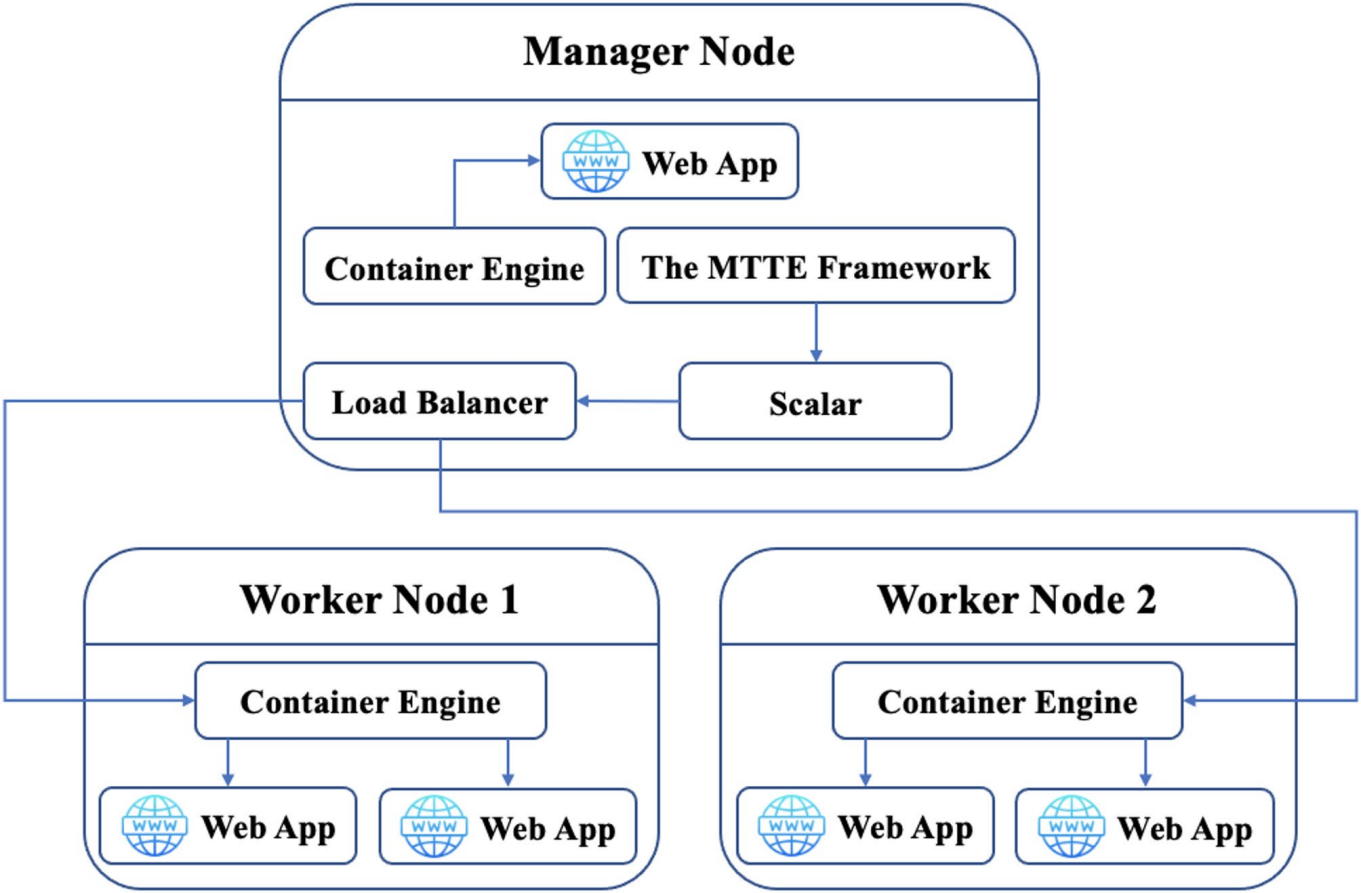
The implemented containerized system used for experimental evaluation.

The experimental environment was implemented using Docker Swarm, a widely used container orchestration platform. A containerized cloud system was deployed to evaluate the performance of the proposed framework, as illustrated in Fig. 5. The environment consists of three interconnected virtual machine nodes running Docker Engine version 20.10.18.

The cluster includes two types of nodes. The manager node is responsible for coordinating the cluster, handling node membership, and managing container deployment, scaling, and load balancing. The worker nodes execute the deployed containers and handle incoming workload requests. The hardware specifications of all cluster nodes are summarized in Table 3.

**Table 3:** Hardware specifications of the Docker cluster nodes.

Node	Memory	Processor	Hard Disk
VM1 (Manager Node)	8 GB	Intel core i7 2 processors /4 cores	1 TB
VM2 (Worker Node)	4 GB	Intel core i7 2 processors / 2 cores	500 GB
VM3 (Worker Node)	4 GB	Intel core i7 2 processors / 2 cores	500 GB

To generate HTTP workloads, an HTTP service was deployed as a containerized application and replicated across worker nodes under the control of the Docker Swarm manager. The service was configured to listen on port 9090, and the manager node across the available containers distributed incoming requests.

The K6 load-testing tool^38^ was used to generate HTTP traffic according to predefined workload profiles, enabling evaluation of the system under different request patterns. Workload data were collected by logging incoming requests over a total duration of 697 min; each record represents the average number of requests per second for a one-minute interval.

The resulting dataset was partitioned into three subsets: training, testing, and deployment. While training and testing sets are used to build and validate the prediction models, the deployment set is used during the continuous operation phase to evaluate prediction accuracy in real time and to determine whether the currently selected model remains the most suitable among the available alternatives.

### Framework implementation

The framework is implemented using Python 3.0. The framework uses three learning algorithms: Auto-Regressive Integrated Moving Average (ARIMA), Random Forest (RF), and Support Vector Machine (SVM). ARIMA is based on the ARMA model and is specified by three parameters: *p*, *d*, and *q*. *p* is the number of lag observations representing the series lag period being fitted for prediction, representing the order of the autoregression polynomial. In contrast, *d* represents the number of times the raw observations are differenced to change the series from non-stationary to stationary. Finally, q signifies the size of the moving average window. Parameters *p*, *d*, and *q* are used by the autoregressive (AR), integrated (I), and moving-average (MA) components of the model. The AR component is an autoregressive model that predicts a variable from its own past values. The integrated part (I) stationaryizes the time series by analyzing differences between observations at different times (I). The moving average (MA) component represents the dependence of an observation on the residual error from a moving average model applied to previous observations.

The SVM model uses support vectors to represent the optimal hyperplane and can solve linear and nonlinear problems. SVM is used in both regression and classification models. It can be applied to time-series forecasting. Still, it needs to transform the dataset into a supervised learning problem using a sliding window of *n* minutes, where each record contains the previous n minutes of workloads and outputs the predicted workload for the next minute. SVM uses a kernel function to map nonlinear inputs into a high-dimensional feature space. SVM is controlled by a group of parameters, which include the kernel function (K), the cost of misclassification (C), the parameter of a Gaussian Kernel to handle non-linear classification (Gamma), and the margin of tolerance (Epsilon).

Random forest is a supervised machine learning algorithm for regression and classification problems. It can be used for time-series forecasting, but, as with SVM, the dataset must be converted into a tabular form compatible with the supervised learning problem. The algorithm constructs multiple decision trees, each trained on a sample from the dataset. The outputs of these trees are then aggregated via voting or averaging. Random forest is controlled by a group of parameters such as the number of decision trees (n_estimators), the maximum number of features used in splitting a node (max_features), the decision tree maximum depth (max_depth), the minimum number of samples required for leaf node (min_samples_leaf), and the minimum number of samples required to split an internal node (min_samples_split).

The parameters of the learning algorithms are selected dynamically, meaning they are adjusted each time an algorithm is trained. The parameters for SVR and Random Forest models are set using grid search^39^. For ARIMA, the model parameters are p, d, and q, denoting the AR, I, and MA terms, respectively. The values are estimated using AutoARIMA. The framework analyzes the Partial Autocorrelation Function (PACF) and Autocorrelation Function (ACF) graphs to fine-tune the model parameters. First, the data are examined for trends or seasonality. If the data exhibit a clear trend, the framework performs differencing until the series becomes stationary, starting with first-order differencing (d = 1) and proceeding to higher orders if necessary. Subsequently, the framework analyzes the PACF graph, which displays the partial autocorrelation at different lags. The framework selects p based on the significant lags in the PACF plot. The framework then analyzes the ACP graph to show autocorrelation across different lags. By examining the ACF plot and identifying the last significant lag before it drops close to zero, the framework can adjust the value of q based on the significant lags in the ACF. After determining d, p, and q, the framework can fit the ARIMA model with these parameters. The values are then validated and adjusted during training. Table 4 presents the automatically adjusted parameter values for the different workloads described in Sect. 4.2.

**Table 4:** Model parameters under different workload patterns.

Model	Parameters	Uniform workload	Linear workload with low rate	Linear workload with a higher rate	Random workload
ARIMA	p	3	9	6	2
	d	2	1	1	1
	q	1	10	3	1
RF	max_depth	110	100	90	90
	max_features	2	5	10	10
	min_samples_leaf	3	1	4	4
	min_samples_split	8	8	8	8
	n_estimators	200	200	100	100
SVR	C	10	10	1	9
	epsilon	0.0001	0.001	0.001	0.001
	gamma	0.01	0.001	0. 1	0.1
	Kernel	rbf	sigmoid	linear	linear

### Experimental results with different workload distribution

**Fig. 6:**
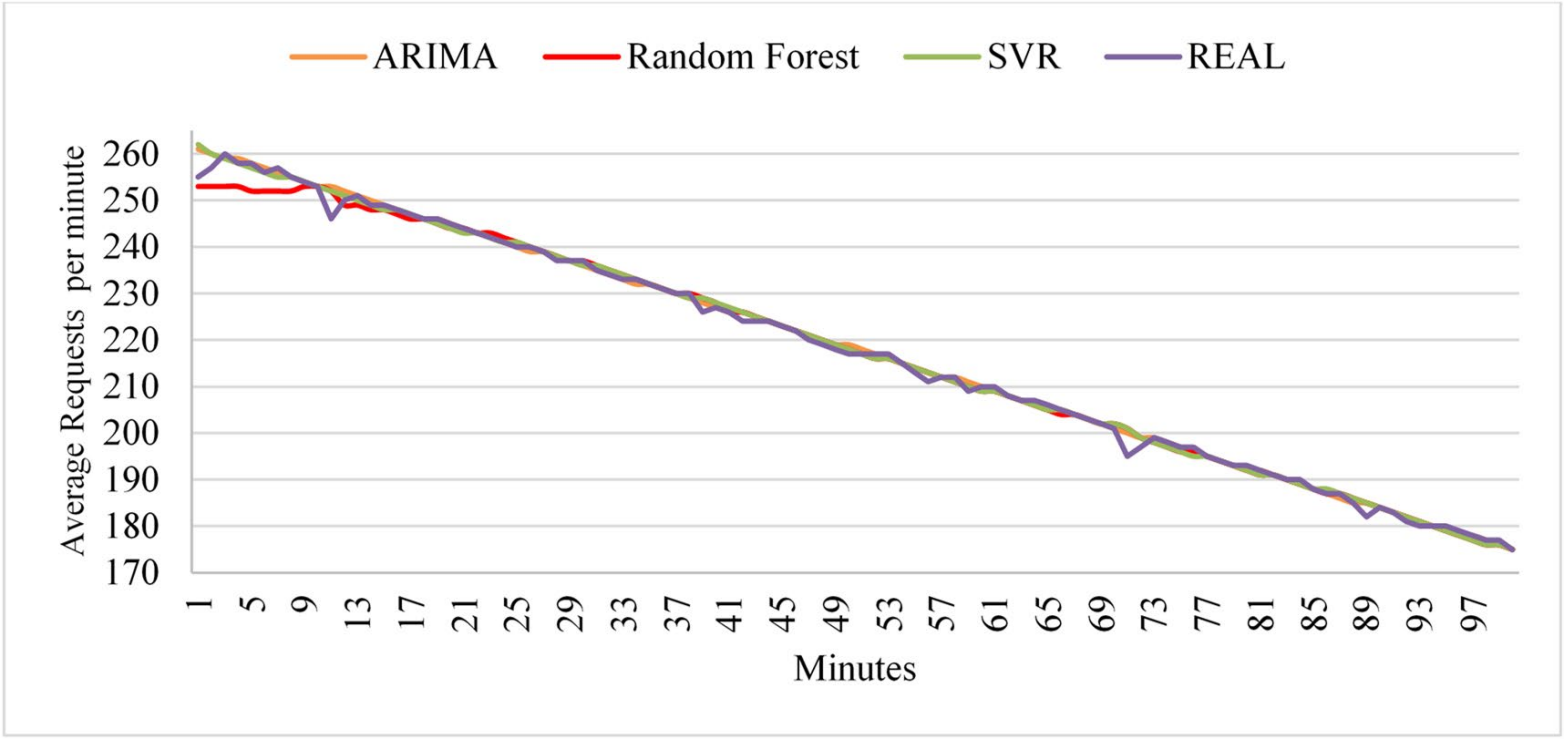
Prediction performance of ARIMA, SVR, and Random Forest under uniform workload conditions during the testing and deployment phases.

The experiments apply the four workload patterns discussed in Sect.  3.1 and evaluate the learning models for each workload pattern. First, we analyze the performance of the three models with uniform workloads. Figure 6 shows the prediction values of the three models during the testing phase. As the figure illustrates, during testing, ARIMA shows strong predictive performance, whereas Random Forest initially shows low accuracy; however, the algorithm begins to demonstrate higher accuracy as the load exhibits more uniform behavior. Since a Random Forest is an ensemble of decision trees, each tree is trained independently and initialized with random data and feature subsets. As a result, the initial predictions from individual trees can be inconsistent and may not generalize well. Additionally, SVR shows higher prediction accuracy than Random Forest. The prediction values of the learning models during the deployment phases indicate that all algorithms maintain their prediction accuracy as observed in the test dataset.

Table 5 also shows evaluation metrics for each algorithm based on prediction results during the testing and deployment phases. The table shows RMSE and MAE along with the performance score. It was found that reporting different evaluation metrics can help provide insights into how the models behave under various conditions. As the results show, the ARIMA algorithm has the optimal score value, which indicates that this algorithm is the best solution for the uniform workload. Additionally, all evaluation metrics yield optimal solutions using the ARIMA algorithm. Based on these results, the framework selects ARIMA as the active model and deploys it to production until the framework triggers the retraining phase. Furthermore, the table shows that during the deployment phases, ARIMA still has the optimal score and evaluation metrics, indicating that the framework made the right decision in selecting ARIMA. Since ARIMA performs better in both phases, the framework chooses ARIMA as the active algorithm for the next deployment cycle.

**Fig. 7:**
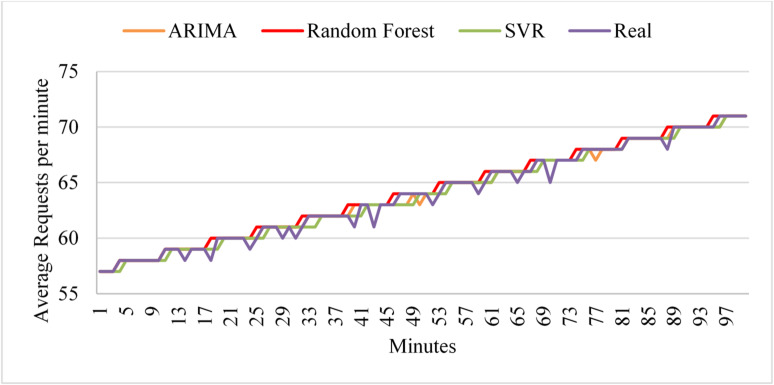
Prediction performance of ARIMA, SVR, and Random Forest under linear workload conditions during the testing phase.

The linear workload represents a steady increase in user requests at a normal rate. Figure 7 shows the predictions of the three models during the test phase. The results indicate that ARIMA exhibits high accuracy, but it fails to predict sudden changes in user requests. In contrast, Random Forest exhibits high prediction accuracy from start to finish, unlike its behavior under the uniform workload (Fig. 6). Similarly, SVR achieves the poorest data fitting compared to the other two algorithms with the linear workload. By the end of the first deployment cycle, the ARIMA score did not exceed the threshold, which led the framework to select it as the active algorithm for the next deployment cycle. Next, if the ARIMA score exceeds the threshold in the next cycle, the framework will pause and retest the threshold before deciding whether to initiate retraining.

**Table 5:** Prediction performance of ARIMA, SVR, and Random Forest under uniform workload conditions for the testing and deployment phases.

	Algorithm	ARIMA	Random Forest	SVR
Test phase	RMSE	1.36	1.8248	1.4662
	MAE	0.67	1.09	0.85
	Score	**0.47%**	0.67%	0.53%
Deployment phase I	RMSE	0.7616	1.7029	0.83
	MAE	0.46	0.8	0.43
	Score	**0.46%**	0.94%	0.47%
Deployment phase II	RMSE	0.7141	1.4866	0.7616
	MAE	0.49	0.73	0.46
	Score	**1.25%**	2.28%	1.27%

Table 6 presents the evaluation results for each model based on the test dataset with a linear workload. As depicted, the ARIMA algorithm achieved the highest score, indicating that it is the best solution for the current workload behavior. Additionally, ARIMA produced the best results for other evaluation metrics. Moreover, it maintained the highest score and evaluation metrics during the subsequent deployment phases. The results demonstrate that ARIMA is the optimal algorithm for the current workload during testing and deployment cycles.

During the deployment phases, all algorithms maintained their prediction accuracy, as seen in the testing phase. However, model performance improves during the second cycle, leading the framework to retain it as the active model. The framework only triggers the training phases when model performance consistently degrades over two successive deployment cycles. As a result, the training process is not repeated frequently, which can consume substantial computational resources. All algorithms improved their prediction performance during the second deployment cycle, with ARIMA remaining the best solution. The algorithm scores shown in the table confirm that continuing to use ARIMA as the active algorithm, without initiating the retraining process, was the right decision.

**Table 6:** Prediction performance of ARIMA, SVR, and random forest under linear workload conditions for the testing and deployment phases.

	Algorithm	ARIMA	Random Forest	SVR
Test phase	RMSE	0.5477	0.6083	0.583
	MAE	0.22	0.27	0.3
	Score	**0.60%**	0.68%	0.69%
Deployment phase I	RMSE	0.8775	0.8544	0.8718
	MAE	0.33	0.39	0.48
	Score	**0.77%**	0.81%	0.86%
Deployment phase II	RMSE	0.7416	0.728	0.728
	MAE	0.33	0.35	0.37
	Score	**0.58%**	0.59%	0.60%

**Fig. 8:**
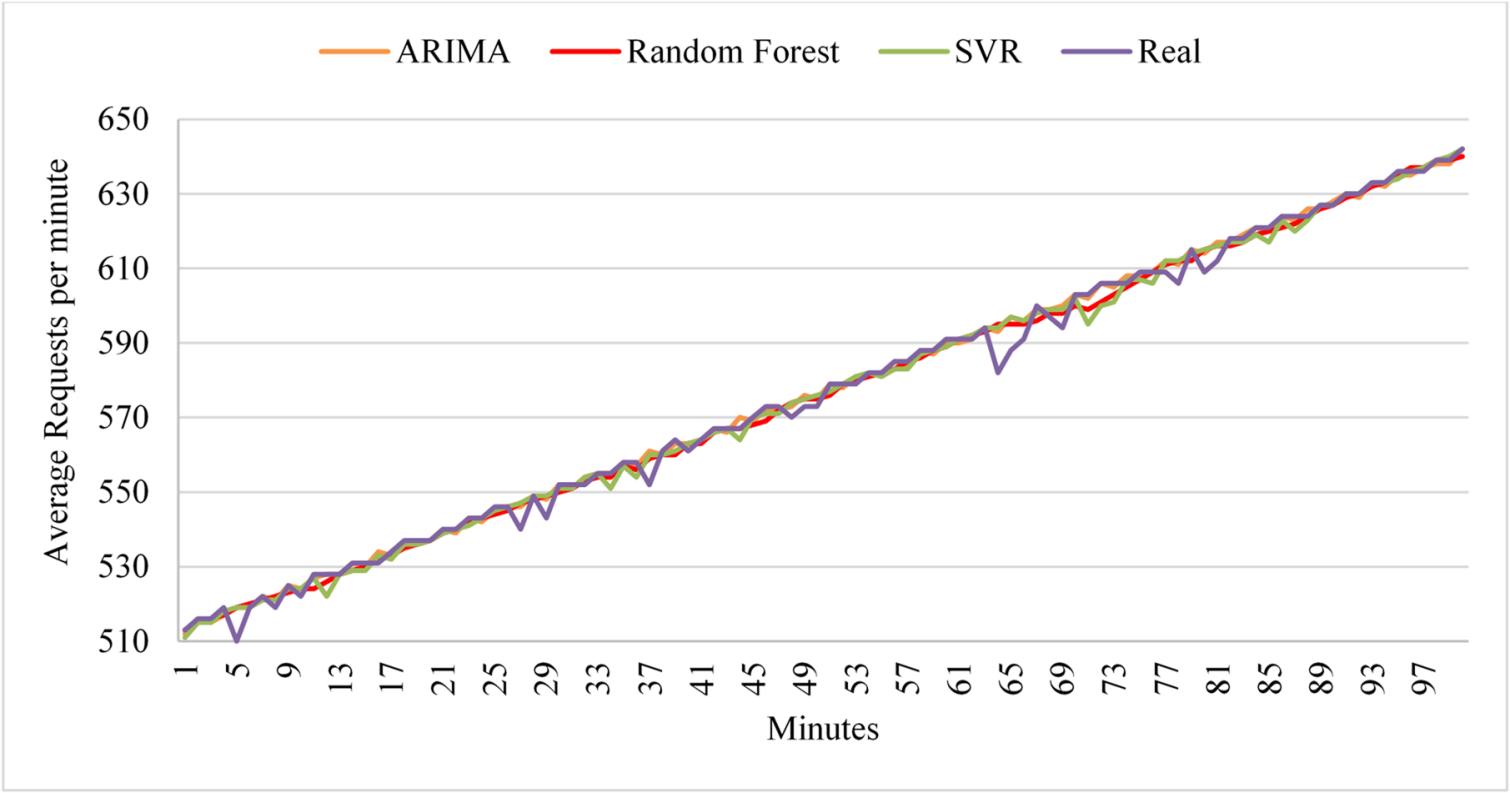
Prediction performance of ARIMA, SVR, and Random Forest under high-rate linear workload conditions during the testing phase.

To evaluate the models with linear workloads at a higher rate, Fig. 8 shows the predictions obtained by the three models on the test dataset. The results indicate that ARIMA achieves high prediction accuracy when the load exhibits uniform behavior. However, it fails to predict sudden changes in user requests. As in its performance with linear workload at a normal rate, the random forest maintains high prediction accuracy throughout the data. Similarly, SVR demonstrates high prediction accuracy as the load increases normally. However, SVR and Random Forest exhibit less curve fitting than ARIMA in this test case.

**Fig. 9:**
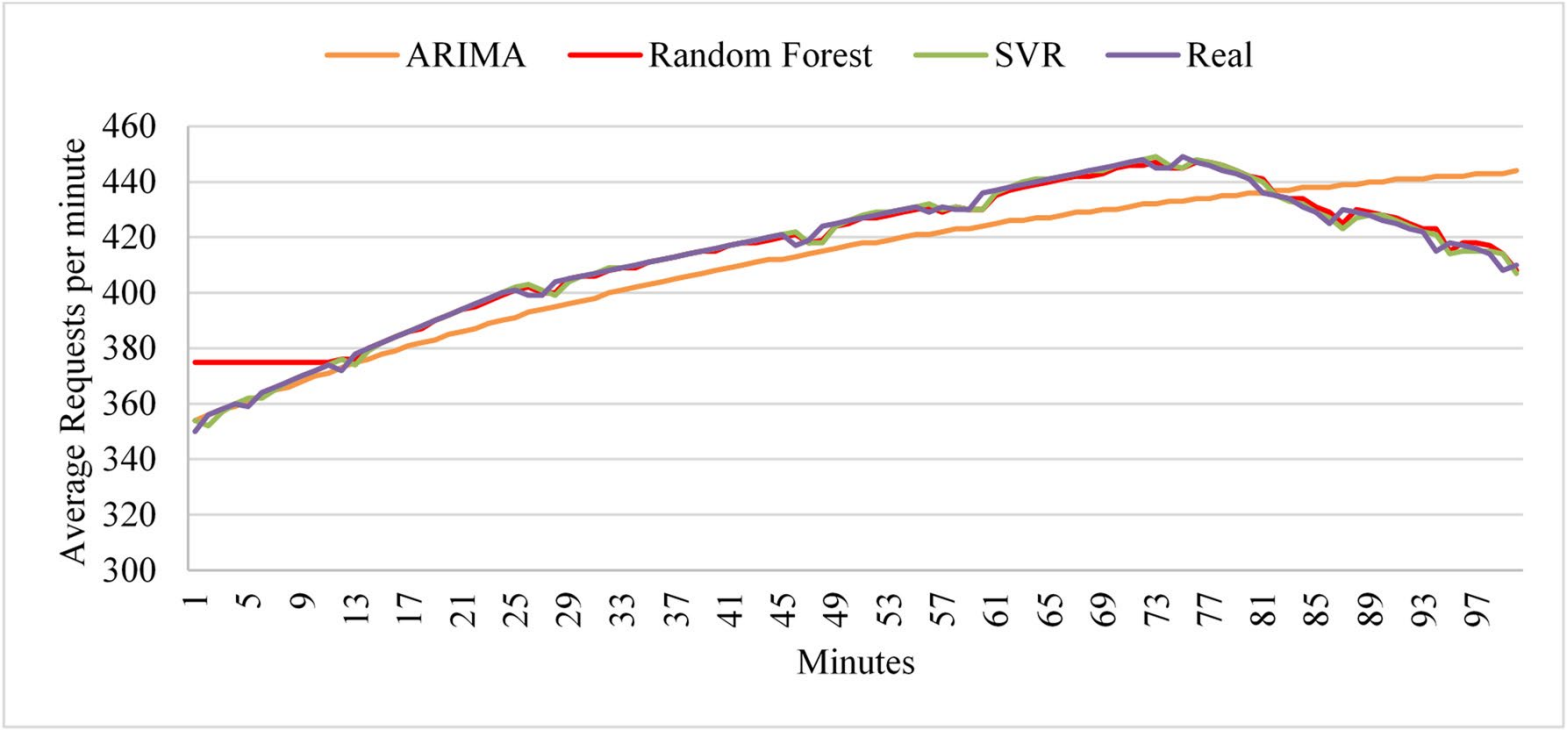
Prediction performance of ARIMA, SVR, and Random Forest under random workload conditions during the testing phase.

Table 7 shows that ARIMA has the optimum score and evaluation metrics. Therefore, the framework chooses ARIMA as the active model for the next deployment cycle with a linear workload at a high rate; Random Forest has higher prediction accuracy than SVR. Additionally, all algorithms maintained their predictive accuracy on the test dataset. However, in the second deployment phase, Random Forest achieved the optimal score, with a very small difference from ARIMA, as in the previous experiment at a normal high rate, resulting in negligible differences in prediction accuracy between Random Forest and ARIMA. ARIMA did not exceed the threshold in either deployment cycle. Random workload represents a non-uniform, unexpected change in user request rate. This is the most common type of workload because users’ behavior is non-uniform and varies over time. Figure 9 shows the prediction results on the test dataset. Random Forest shows low prediction accuracy initially, but performance improves; at the peak, when requests begin to change behavior and decrease, accuracy rises. On the other hand, SVR achieves high accuracy from the outset, even with a curve change at the peak. Also, Random Forest shows consistent prediction performance, especially with more data. Also, ARIMA shows high predictive accuracy initially, but after a very short time, it shows poor accuracy.

**Table 7:** Prediction performance of ARIMA, SVR, and Random Forest under high-rate linear workload conditions for the testing and deployment phases.

	Algorithm	ARIMA	Random forest	SVR
Test phase	RMSE	2.6192	2.9155	3.1591
	MAE	1.5	1.98	2.1
	Score	**0.36%**	0.42%	0.45%
Deployment phase I	RMSE	2.74	3.1177	3.985
	MAE	1.83	2.14	2.13
	Score	**0.32%**	0.37%	0.39%
Deployment phase II	RMSE	2.7258	2.91	3.1953
	MAE	1.95	1.7	1.73
	Score	0.28%	**0.27%**	0.29%

The results show that SVR achieves the highest performance on random workloads, as reflected by the evaluation metrics reported in Table 8. In contrast, Random Forest exhibits moderate accuracy, while ARIMA performs poorly under highly irregular request patterns. Based on these results, the framework selects SVR as the active model for the subsequent deployment cycle, with its hyperparameters automatically tuned during training. The selected model is then evaluated on the deployment dataset to verify that its predictive quality is maintained under operational conditions.

During the deployment phase, SVR continues to deliver the most stable and accurate predictions across random workload segments. Although Random Forest achieves comparable performance in some intervals, it remains consistently less accurate than SVR, while ARIMA shows persistent degradation. Since the SVR performance does not exceed the predefined retraining threshold, it remains selected for the next deployment cycle, avoiding unnecessary retraining.

**Table 8:** Prediction performance of ARIMA, SVR, and Random Forest under random workload conditions.

	Algorithm	ARIMA	Random Forest	SVR
Test phase	RMSE	11.952	4.9629	2.1656
	MAE	9.85	2.59	1.27
	Score	2.63%	0. 91%	**0. 41%**
Deployment phase I	RMSE	21.82	2.17	2.1
	MAE	16.76	1.57	1.45
	Score	5.54%	0.54%	**0.51%**
Deployment phase II	RMSE	83.57	7.01	6.3984
	MAE	67.98	3.63	1.92
	Score	21.90%	1.54%	**1.20%**

Across all workload types, the three models exhibit distinct strengths. ARIMA provides the highest accuracy for uniform and smooth linear workloads, where temporal patterns are stable and predictable. SVR performs competitively in these scenarios but is slightly less accurate. For random workloads, SVR consistently outperforms both ARIMA and Random Forest, while Random Forest offers reasonable but inferior performance.

Each experiment was repeated 20 times to ensure stability of the results. Variance across runs was negligible for the controlled synthetic workloads and is therefore omitted for clarity. For experiments using real-world datasets, standard deviation values will be reported to reflect variability under realistic operating conditions.

### Applying the proposed model on a real dataset

After evaluating the framework using simulated workload patterns and verifying its ability to select the most suitable prediction model, the framework was further validated using real-world workload data. To ensure the reliability of the results, each experiment was repeated 20 times for each dataset. The reported performance values represent the average across all runs, while the corresponding standard deviation is included to quantify variability between trials.

The parameter settings used for the FIFA World Cup 2018 workload dataset are summarized in Table 9, including the automatically selected configurations for ARIMA, Random Forest, and SVR models.

**Table 9:** Model parameter settings for the FIFA World Cup 2018 workload dataset.

Model	Parameters	Value
ARIMA	p	8
	d	1
	q	10
RF	max_depth	40
	max_features	10
	min_samples_leaf	1
	min_samples_split	12
	n_estimators	100
SVR	C	10
	epsilon	0.1
	gamma	0.001
	Kernel	rbf

The dataset consists of 29 days of hourly workload measurements (696 records, 29 × 24) collected from the FIFA World Cup 2018 website between May 1 and May 29, 2018, representing the number of user requests. The training dataset covers the first 10 days (May 1–10). This training window was selected based on preliminary experiments, which showed that using long training periods reduced prediction accuracy due to the highly dynamic and rapidly changing request patterns throughout the day.

The testing phase was set to 24 h in order to capture the full range of daily workload variations, as request patterns were observed to repeat on a daily basis. Despite this periodicity, the workload remains classified as random because of frequent sudden increases and decreases in request rates.

To evaluate the long-term stability of the model-selection mechanism, the framework was tested over multiple consecutive deployment cycles. As in the previous experiments, model parameters were determined automatically during the training phase. The resulting parameter configurations and performance metrics obtained during the testing and subsequent deployment stages are summarized in Table 10.

**Fig. 10:**
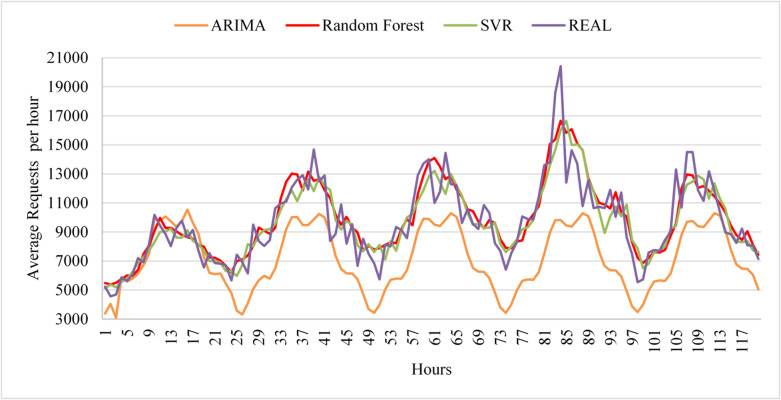
Prediction performance of ARIMA, SVR, and Random Forest on the FIFA World Cup 2018 workload across the testing phase and successive deployment cycles.

Figure 10 presents the prediction results of all three models over the testing phase and four deployment cycles. Random Forest achieves the highest overall prediction accuracy, with only minor fluctuations across cycles that remain below the retraining threshold. SVR also shows strong performance but remains consistently less accurate than Random Forest throughout the deployment phases. In contrast, ARIMA exhibits the lowest prediction accuracy among the three models for this dataset.

The results show that Random Forest achieves the highest performance scores under random workload conditions, indicating that it is the most suitable model for this workload type. SVR also demonstrates good predictive accuracy, but it remains consistently less accurate than Random Forest, while ARIMA performs poorly for highly irregular workloads. Based on these results, the framework selects Random Forest as the active model for the next deployment cycle, with its hyperparameters automatically tuned during training. The selected model is then evaluated on the deployment dataset to verify that its performance is maintained under operational conditions.

Across subsequent deployment cycles, Random Forest continues to achieve the highest scores and evaluation metrics, confirming the stability of the model selection. Since its performance remains above the predefined threshold, retraining is not triggered, allowing the framework to maintain high prediction accuracy while minimizing computational overhead. The gradual improvement in performance over successive cycles is illustrated in Fig. 12.

After validating the framework on real-world data using ARIMA, SVR, and Random Forest, a further experiment was conducted in which ARIMA was replaced by Long Short-Term Memory (LSTM), as ARIMA showed limited effectiveness for random workloads. LSTM is a recurrent neural network designed to capture long-term dependencies in sequential data, making it well suited for time-series forecasting tasks. By incorporating LSTM into the model pool, the framework was further tested under more challenging workload conditions using a neural-network-based predictor.

### Evaluating the model with changes in the used algorithms

The proposed framework integrates both traditional and learning-based prediction models. Classical approaches such as ARIMA, SVR, and Random Forest were included because they provide reliable and computationally efficient baselines suitable for real-time deployment. In addition, a Long Short-Term Memory (LSTM) model is evaluated in this section to demonstrate that the framework is not restricted to conventional machine-learning methods, but can also incorporate neural-network-based predictors when deeper temporal modeling is required. The modular design of the proposed MTTD framework allows prediction models—including LSTM and other sequence models—to be added, replaced, or removed according to workload characteristics and available computational resources.

The purpose of this experiment is to evaluate the ability of the framework to operate with different types of prediction models while maintaining low overhead and high accuracy. To balance performance and computational cost, a maximum of three models is used at any time. The experiment was conducted using workload data from the FIFA World Cup 1998 website, which recorded the average number of user requests per second at one-minute intervals. The dataset consists of 1440 min (24 h) of activity, covering the period from May 1, 22:00 to May 2, 21:59.

**Table 10:** Prediction performance of the models over the testing phase and four deployment cycles using the FIFA World Cup 2018 workload.

	Algorithm	ARIMA	Random Forest	SVR
Test phase	RMSE	991.672	645.386	701.489
	MAE	798.75	516.625	556.417
	Score	12.10%$\:\pm\:$0.03	**7.80%** $\:\pm\:$ **0.01**	8.50%$\:\pm\:$0.02
Deployment phase I	RMSE	3024.682	1310.291	1382.086
	MAE	2816.417	1110.833	1104.375
	Score	29%$\:\pm\:$0.04	**12%** $\:\pm\:$ **0.01**	12.40%$\:\pm\:$0.03
Deployment phase II	RMSE	3209.715	1264.078	1353.631
	MAE	3051.542	1010.958	1111.792
	Score	1.$\:\pm\:$0.04	**11%** $\:\pm\:$ **0.02**	11.90%$\:\pm\:$0.01
Deployment phase III	RMSE	4813.245	1750.127	1947.022
	MAE	4352.625	1348.542	1398.5
	Score	39.10%$\:\pm\:$0.1	**13.20%** $\:\pm\:$ **0.01**	14.20%$\:\pm\:$0.01
Deployment phase IV	RMSE	2756.046	1182.378	1359.781
	MAE	2415.625	898.083	1007.833
	Score	26.60%$\:\pm\:$0.05	**10.70%** $\:\pm\:$ **0.01**	12.20%$\:\pm\:$0.02

Training data were taken from May 1, with the size of the training set determined empirically. Since request rates fluctuate rapidly within short time intervals, using excessively long training periods was found to reduce prediction accuracy. The testing phase was set to 100 min, which captures the main variations in request patterns over approximately one and a half hours. The workload is classified as random because of frequent and abrupt changes in request rates during this period. Table 11 summarizes the model parameters used for the FIFA World Cup 1998 dataset.

To further evaluate the adaptability of the framework, the algorithm was tested with additional prediction models to examine its ability to detect performance degradation and trigger retraining when necessary. For this purpose, deployment cycles were allowed to continue until the retraining condition was met. As in previous experiments, model parameters were determined automatically during the training phase. The resulting parameter values and performance metrics obtained during the testing phase and subsequent deployment stages are reported in Table 12.

**Table 11:** Model parameter settings for the FIFA World Cup 1998 workload dataset.

Model	Parameters	Value
LSTM	Call backs	Early stop
	Dropout	0.2
	Activation	relu
	Loss	Mean square error
	Optimizer	adam
	Neurons	20
	Epochs	100
	Features	5
RF	Max_depth	80
	Max_features	5
	Min_samples_leaf	5
	Min_samples_split	12
	n_estimators	100
SVR	C	2
	Epsilon	1
	Gamma	0.1
	Kernel	rbf

**Fig. 11:**
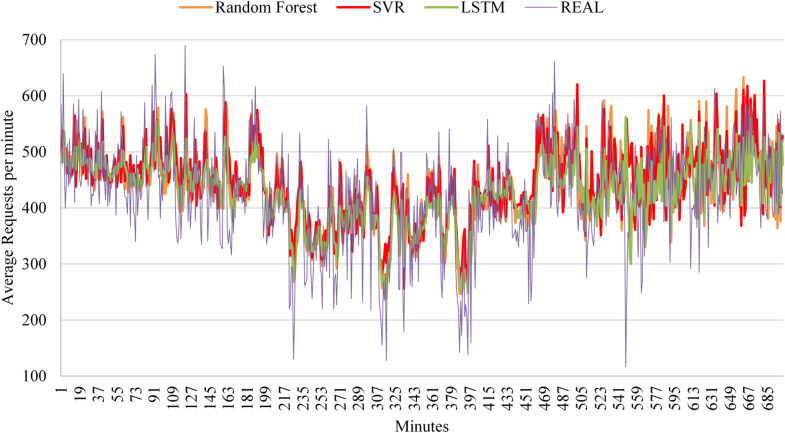
Evolution of prediction scores across successive deployment cycles under random workload conditions.

After the testing phase, the framework selected LSTM as the active model, as it achieved the highest score, as reported in Table 12. In the subsequent deployment cycles, LSTM continued to provide the best overall performance, confirming the validity of the initial selection. This behavior is illustrated in Fig. 11, except for the fourth deployment cycle, where Random Forest achieved a slightly higher score.

**Table 12:** Prediction performance over the testing phase and the first six deployment cycles for the FIFA World Cup 1998 workload.

	Algorithm	Random Forest	SVR	LSTM
Test phase	RMSE	66.861	68.99	62.8387
	MAE	53.93	55.4	50.64
	Score	12.60%$\:\pm\:$0.02	13%$\:\pm\:$0.01	**11.90%** $\:\pm\:$ **0.01**
Deployment phase I	RMSE	75.557	78.266	75.139
	MAE	60.18	62.95	59.01
	Score	14.81%$\:\pm\:$0.03	15.40%$\:\pm\:$0.02	**14.60%** $\:\pm\:$ **0.02**
Deployment phase II	RMSE	64.584	68.462	60.845
	MAE	51.46	54.35	48.83
	Score	15.30%$\:\pm\:$0.02	16.20%$\:\pm\:$0.03	**14.50%** $\:\pm\:$ **0.02**
Deployment phase III	RMSE	71.92	76.38	66.513
	MAE	58.66	61.52	54.86
	Score	18.20%$\:\pm\:$0.04	19.20%$\:\pm\:$0.04	**16.90%** $\:\pm\:$ **0.03**
Deployment phase IV	RMSE	71.383	75.646	71.908
	MAE	56.22	59.18	57.72
	Score	**14.40%** $\:\pm\:$ **0.03**	15.30%$\:\pm\:$0.02	14.70%$\:\pm\:$0.02
Deployment phase V	RMSE	106.093	109.527	105.935
	MAE	81.36	81.64	79.39
	S core	21.50%$\:\pm\:$0.04	21.90%$\:\pm\:$0.03	**21.30%** $\:\pm\:$ **0.03**
Deployment phase VI	RMSE	105.669	101.739	95.129
	MAE	86.45	82.43	77.17
	S core	20.40%$\:\pm\:$0.04	19.60%$\:\pm\:$0.03	**18.30%** $\:\pm\:$ **0.03**

During the fifth deployment cycle, the LSTM score exceeded the predefined retraining threshold, which triggered a threshold adjustment to allow one additional evaluation cycle. However, since the score again exceeded the threshold in the following cycle, the framework initiated retraining at the end of the sixth deployment cycle. The retrained models were then evaluated during the testing phase in preparation for the seventh deployment cycle.

Following retraining, SVR achieved the highest performance score and was therefore selected as the new active model. SVR continued to outperform the other models in the subsequent deployment cycles, as summarized in Table 13. Its performance improved consistently across cycles, exceeding the scores obtained before retraining, as illustrated in Figs. 13 and 14.

A comparison between continuing with the previously selected LSTM model and switching to the newly selected SVR model after retraining demonstrates a clear improvement in prediction accuracy. This improvement is shown in Fig. 15, highlighting the benefit of resetting the active model when performance degradation is detected.

**Table 13:** Prediction performance over the first four retrained deployment cycles for the FIFA World Cup 1998 workload.

	Algorithm	Random Forest	SVR	LSTM
Test phase	RMS	69.429	65.481	68.077
	MAE	56.04	53.19	54.9
	Score	13.30%$\:\pm\:$0.03	**12.60%** $\:\pm\:$ **0.02**	13.10%$\:\pm\:$0.03
Deployment phase I	RMS	127.223	120.882	128.358
	MAE	103.72	94.26	101.68
	Score	18.80%$\:\pm\:$0.03	**17.40%** $\:\pm\:$ **0.02**	18.70%$\:\pm\:$0.03
Deployment phase II	RMS	129.221	127.463	135.819
	MAE	97.85	97.03	104.26
	Score	17.30%$\:\pm\:$0.02	**17.10%** $\:\pm\:$ **0.02**	18.30%$\:\pm\:$0.03
Deployment phase III	RMS	145.39453	142.16329	142.96276
	MAE	118.11	116.92	150.67511
	Score	17.99%$\:\pm\:$0.04	**17.69%** $\:\pm\:$ **0.03**	20.10%$\:\pm\:$0.04
Deployment phase IV	RMS	134.555	129.248	133.832
	MAE	109.78	105.64	108.39
	Score	16.10%$\:\pm\:$0.02	**15.50%** $\:\pm\:$ **0.02**	16%$\:\pm\:$0.03

**Fig. 12:**
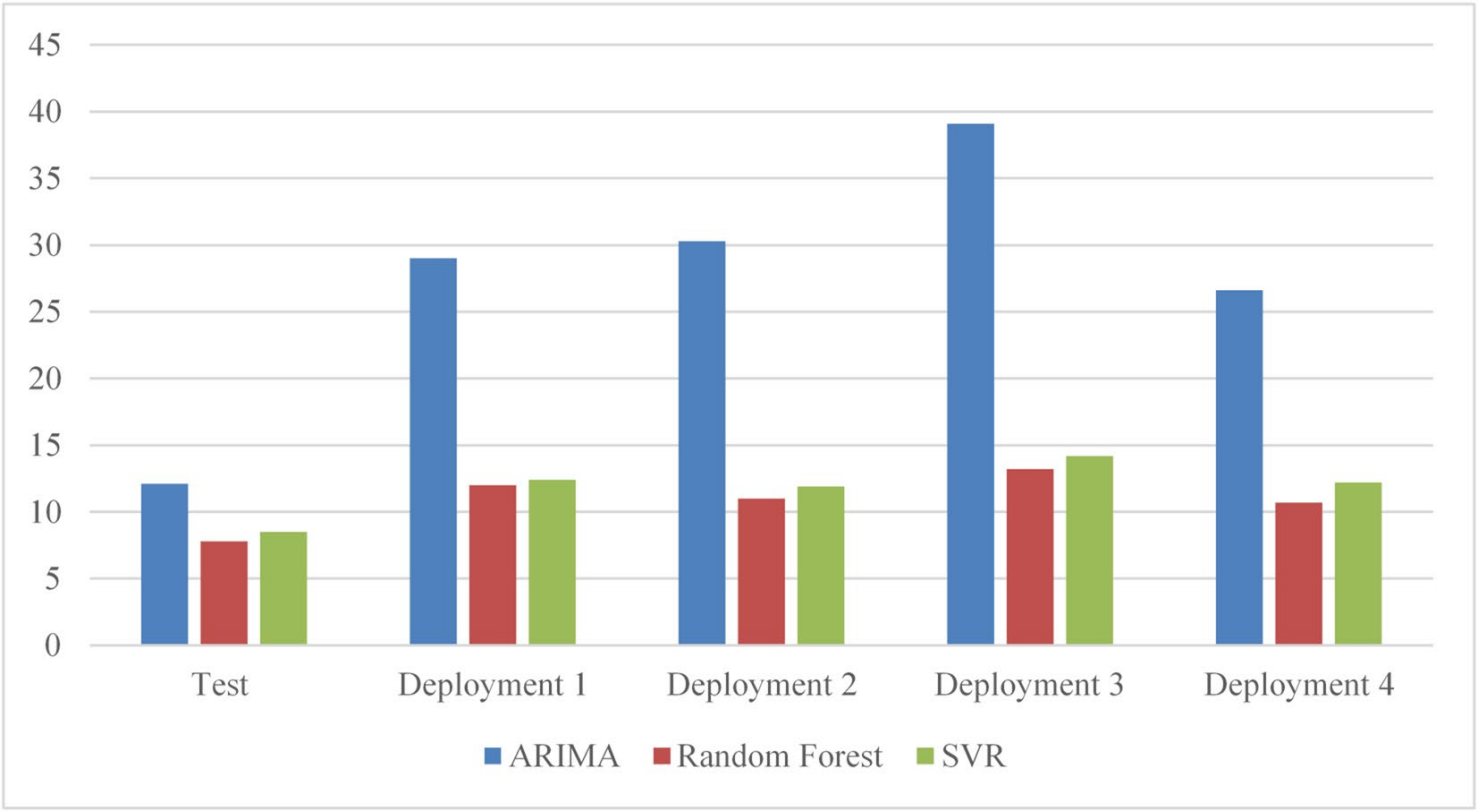
Score trends for the FIFA World Cup 2018 workload across deployment cycles.

**Fig. 13:**
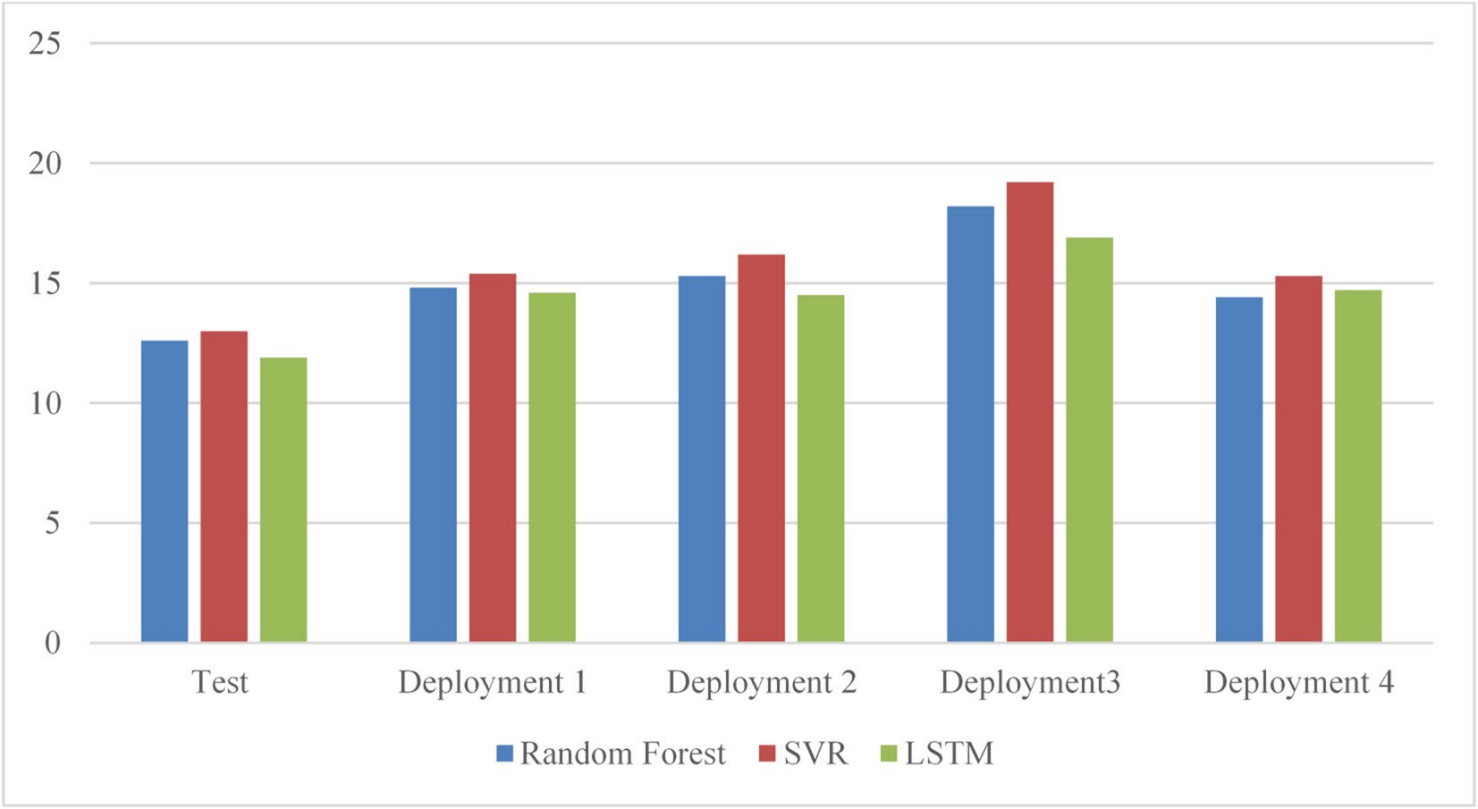
Score trends for the FIFA World Cup 1998 workload during the first four deployment cycles.

**Fig. 14:**
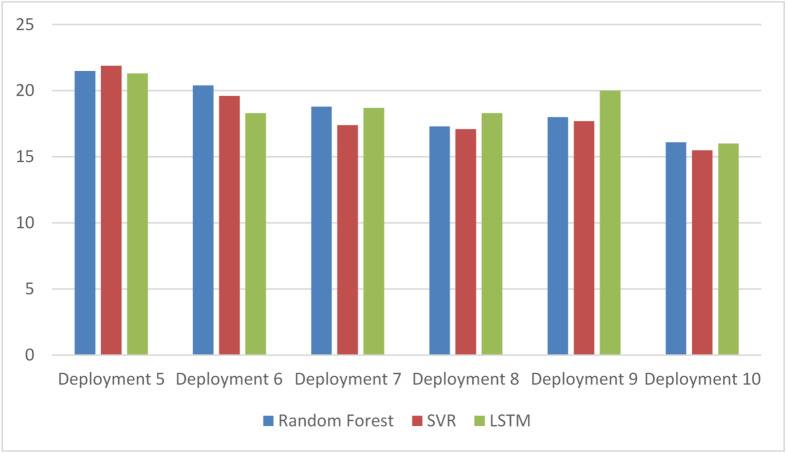
Score trends for the FIFA World Cup 1998 workload during retraining cycles.

**Fig. 15:**
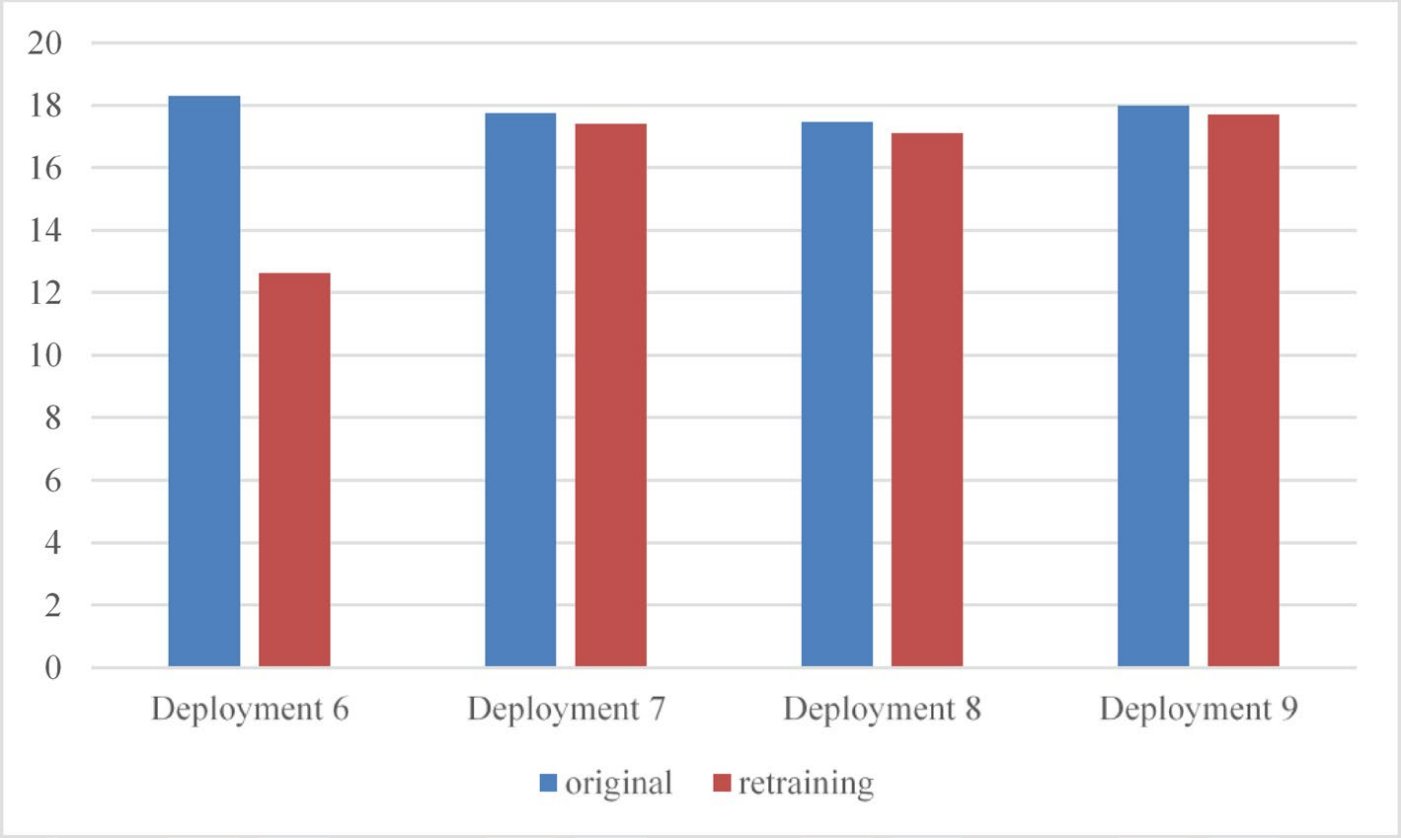
Comparison of prediction scores for the FIFA World Cup 1998 workload before and after retraining across deployment cycles.

## Conclusion and future work

This study addressed the challenge of highly dynamic and heterogeneous HTTP workload patterns in cloud environments by introducing a Monitor–Train–Test–Deploy (MTTD) framework that automatically selects and switches among multiple prediction models based on workload behavior and real-time performance. The experimental results demonstrate that no single algorithm can consistently achieve optimal accuracy across all traffic conditions. ARIMA performs best under stable and regular workloads, while SVR, Random Forest, and particularly LSTM achieve superior accuracy under highly variable and bursty traffic patterns.

By dynamically selecting the most suitable model at each stage of operation, the proposed framework significantly improves prediction robustness and adaptability compared with static single-model approaches, while maintaining efficient time and resource usage through selective model activation. The results further show that the framework can sustain high prediction accuracy across multiple deployment cycles without unnecessary retraining, thereby reducing computational overhead in production environments.

The modular and extensible design of the MTTD framework enables prediction models to be added, replaced, or removed without modifying the system structure, ensuring long-term adaptability to evolving workload characteristics and emerging learning techniques. Although the current implementation focuses on univariate workload prediction, the framework can be extended to incorporate additional system metrics such as CPU usage, memory consumption, and network traffic.

Future work will integrate the MTTD framework with container auto-scaling mechanisms and evaluate its performance in large-scale real-world cloud platforms. Further studies will also explore the inclusion of advanced sequence models, including Transformer-based and Informer-based predictors, as well as multivariate workload modeling, to enhance prediction accuracy under increasingly complex cloud workloads.

## Data Availability

The datasets analyzed during the current study are available in the Workload-prediction- repository, https://github.com/walidmoussa2010/Workload-prediction-.
